# Many people admitted to hospital with a provisional diagnosis of nonserious back pain are subsequently found to have serious pathology as the underlying cause

**DOI:** 10.1007/s10067-022-06054-w

**Published:** 2022-01-11

**Authors:** Alla Melman, Chris G. Maher, Chris Needs, Gustavo C. Machado

**Affiliations:** 1 Institute for Musculoskeletal Health, Sydney Local Health District and University of Sydney, Level 10N, King George V Building, Royal Prince Alfred Hospital PO Box M179, Missenden Road Camperdown, NSW 2050 Sydney, Australia; 2 Rheumatology Department, Royal Prince Alfred Hospital, Sydney, NSW Australia

**Keywords:** Back pain, Nonserious back pain, Spinal, Spinal pathology

## Abstract

To determine the proportion of patients admitted to the hospital for back pain who have nonserious back pain, serious spinal, or serious other pathology as their final diagnosis. The proportion of nonserious back pain admissions will be used to plan for future ‘virtual hospital’ admissions. Electronic medical record data between January 2016 and September 2020 from three emergency departments (ED) in Sydney, Australia were used to identify inpatient admissions. SNOMED-CT-AU diagnostic codes were used to select ED patients aged 18 and older with an admitting diagnosis related to nonserious back pain. The inpatient discharge diagnosis was determined from the primary ICD-10-AM codes by two independent clinician-researchers. Inpatient admissions were then analysed by sociodemographic and hospital admission variables. A total of 38.1% of patients admitted with a provisional diagnosis of nonserious back pain were subsequently diagnosed with a specific pathology likely unsuitable for virtual care; 14.2% with a serious spinal pathology (e.g., fracture and infection) and 23.9% a serious pathology beyond the lumbar spine (e.g., pathological fracture and neoplasm). A total of 57% of admissions were identified as nonserious back pain, likely suitable for virtual care. A challenge for implementing virtual care in this setting is screening for patients with serious pathology. Protocols need to be developed to reduce the risk of patients being admitted to virtual hospitals with serious pathology as the cause of their back pain.

**Table:** 

**Key Points** *• Among admitted patients provisionally diagnosed in ED with non-serious back pain, 38.1% were found to have ‘serious spinal pathologies’ or ‘serious pathologies beyond the lumbar spine’ at discharge.* *• Spinal fractures were the most common serious spinal pathology, accounting for 9% of all provisional ‘non-serious back pain’ admissions from ED.* *• 57% of back pain admissions were confirmed to be non-serious back pain and may be suitable to virtual hospital care; the challenge is discriminating these patients from those with serious pathology.*

**Supplementary Information:**

The online version contains supplementary material available at 10.1007/s10067-022-06054-w.

## Introduction

Guidelines for managing low back pain recommend that it should be managed in primary care [1], however increasingly, patients are presenting to the ED. For example, in the US, Canada and Australia [2, 3], low back pain ranks in the top 10 reasons for an ED presentation. Many of those who present to ED are subsequently admitted as an inpatient. Two recent Australian studies reported that 18 [4] and 53% [5] were admitted as inpatients. The first study estimated that the cost of each admission was ~ AUD$15,000 [6].

Development of clinical pathways allowing for avoidance of ‘traditional admission’ is urgently needed. A potential solution is to offer a ‘virtual hospital’ model of care to select patients who would do well with this approach [7]. The virtual hospital model of care is based on a hybrid ‘hospital in the home’ model of care, incorporating home visits and remote monitoring. Careful patient selection would be key to the success of this approach [8, 9]; one consideration would be excluding those with serious conditions that would be best managed in an inpatient setting.

Estimates for the prevalence of serious spinal pathology in patients presenting with back pain to the ED are higher than those for patients presenting to primary care. A systematic review of 22 studies in the ED reported rates of 2.5 to 5.1% in prospective and 0.7 to 7.4% in retrospective studies for pathologies requiring immediate or urgent treatment [10]. These estimates are consistent with a subsequent Australian study that found 4.5% of all back pain presentations to ED were diagnosed as serious spinal pathologies [4]. These rates contrast with rates of serious pathology of ~ 1% for LBP patients presenting to primary care [1].

At present, no study has focussed on the discharge diagnoses of patients who were admitted, via the ED, with a provisional diagnosis of nonserious back pain. Understanding the prevalence and profile of these patients may lead to a better understanding of the feasibility of a virtual hospital model care as an alternative to inpatient admission for back pain. Thus, in this study, we determined the proportion of patients admitted with nonserious back pain who received a discharge diagnosis of serious spinal or non-spinal pathologies or other musculoskeletal conditions. We also examined the specific pathologies within these broad categories and the patient characteristics associated with these categories.

## Methods

We followed the Reporting of studies Conducted using Observational Routinely-collected health Data (RECORD) statement [11]. Approval was granted by the Sydney Local Health District (SLHD) Human Research Ethics Committee (protocol X20-0362).

We accessed electronic medical record data for the three SLHD EDs between January 2016 and September 2020. SNOMED-CT-AU diagnostic codes were used to select ED patients aged 18 and older with an admitting diagnosis related to nonserious back pain (i.e., nonspecific back pain or radicular back pain) [4]. The discharge diagnosis was determined from the discharge primary ICD-10-AM codes. These codes were collapsed into the following five broad categories:

i. nonspecific back pain
ii. radicular back pain
iii. serious spinal pathology
iv. serious pathology beyond the lumbar spine
v. other musculoskeletal conditions (nonserious, beyond lumbar spine)

Categorisation of primary ICD-10-AM codes was completed independently by two experienced clinicians, and any disagreements were resolved by consensus reviewing the inpatient electronic medical record of such patients. ICD-10-AM sub-categories were created for two categories: ‘serious spinal pathologies’ and ‘pathology beyond the lumbar spine’, to ascertain the prevalence of specific pathologies.

Socio-demographic variables such as age, sex, and postcode were collected. Patient postcode was linked to SEIFA (Socio-Economic Indexes for Areas) categories and clustered by deciles 1–5 and 6–10, as a proxy for socioeconomic status. Data regarding ED admission included mode of arrival (ambulance or non-ambulance), and Australasian triage scale category (1–5). Hospital length of stay in the ED as well as on the ward was also collected.

## Results

We included 1982 admissions from ED with a provisional diagnosis of nonserious back pain. These admissions were coded to 394 unique ICD-10-AM diagnostic codes and distributed to the five broad categories (see Table 1).

**Table 1:** Prevalence of discharge diagnostic categories in patients admitted with a provisional diagnosis of nonserious back pain, by age group

Back pain category		Total (*n* = 1982)	Age < 65 (*n* = 708)	Age ≥ 65 (*n* = 1274)
Nonspecific back pain, *n* (%)		543 (27.4)	209 (29.5)	334 (26.2)
Radicular back pain, *n* (%)		586 (29.6)	257 (36.3)	329 (25.8)
Serious spinal pathology, *n* (%)	Fracture	172 (8.7)	75 (10.6)	206 (16.2)
	Infection	42 (2.1)		
	Cauda equina, myelopathy, spinal cord compression/injury	20 (1.0)		
	Postsurgical or procedural complication	15 (0.8)		
	Inflammatory spondylopathy	13 (0.7)		
	Osteoporotic fracture	7 (0.4)		
	Dislocation of thoracolumbar vertebrae	6 (0.3)		
	Neurological condition	4 (0.2)		
	Neoplasm	2 (0.1)		
	Total	281 (14.2)		
Serious pathology beyond lumbar spine, *n* (%)	Pathological fracture	145 (7.3)	134 (18.9)	339 (26.6)
	Infection	86 (4.3)		
	Neoplasm	56 (2.8)		
	Trauma (contusion, wound, or fracture)	40 (2.0)		
	Neurological condition	28 (1.4)		
	Cardiovascular condition	23 (1.2)		
	Gastroenterological condition	23 (1.2)		
	Metabolic disorder	12 (0.6)		
	Urological condition	12 (0.6)		
	Delirium or dementia	11 (0.6)		
	Myeloma	9 (0.5)		
	Inflammatory arthropathy	7 (0.4)		
	Gynaecological condition	7 (0.4)		
	Lymphoma	7 (0.4)		
	Leukemia	4 (0.2)		
	Respiratory condition	3 (0.2)		
	Total	473 (23.9)		
Other musculoskeletal condition (non-serious, non-lumbar), *n* (%)		99 (5.0)	33 (4.7)	66 (5.2)

A total of 1129/1982 (57%) of admissions were identified as nonserious back pain, likely suitable for virtual care. These patients had a mean age of 70 [51–82], 41% were female, with 73.4% living in areas with a higher average socioeconomic index (6–10). Postcode data were missing for 30 admissions. 61.6% of nonserious back pain patients arrived by ambulance, with 53% being triaged as ATS 3, classified as severe pain (see Table 2).

**Table 2:** Characteristics of admitted patients likely suitable (nonserious back pain) and likely unsuitable (serious spinal or non-lumbar pathology) for virtual hospital care

Characteristics	Total, *n* (%) 1982 (100)	Nonserious back pain, *n* (%) 1129 (57)	Serious spinal or non-lumbar pathology, *n* (%) 853 (43)
Age, median [IQR]	73 [56–83]	70 [51–82]	76 [63–84]
Female sex, *n* (%)	1189 (60.0)	463 (41.0)	330 (38.7)
Socioeconomic indexes for areas, *n* (%)			
*SEIFA 1–5*	510 (26.1)	296 (26.6)	214 (25.5)
*SEIFA 6–10*	1442 (73.9)	815 (73.4)	627 (74.6)
ED arrival by ambulance, *n* (%)	1235 (62.3)	695 (61.6)	540 (63.3)
#ED triage category, *n* (%)			
2	63 (3.2)	34 (3.0)	29 (3.4)
3	1060 (53.5)	601 (53.2)	459 (53.8)
4	850 (42.9)	489 (43.3)	361 (42.3)
5	9 (0.5)	5 (0.4)	4 (0.5)
ED length of stay (hours), median [IQR]	6 [4–8]	5 [4–7]	6 [4–8]
Inpatient length of stay (days), median [IQR]	6 [3–12]	4 [2–8]	8 [4–16]

^**#**^**ED Triage categories: 2:** Imminently life-threatening; **3:** potentially life-threatening or important time-critical treatment or severe pain; **4:** potentially life-serious or situational urgency or significant complexity; **5:** less urgent

However, a significant proportion of patients admitted with nonserious back pain were subsequently diagnosed with a specific pathology likely unsuitable for virtual care; 14.2% with a serious spinal pathology and 23.9% a serious pathology beyond the lumbar spine. The most common serious spinal pathologies were fracture (8.7%) and infection (2.1%), and the most common serious pathologies beyond the spine were pathological fracture (7.3%) and infection (4.3%).

In those aged ≥ 65, serious spinal pathology had a prevalence rate of 16.2%, compared to 10.6% in those under 65. Pathologies beyond the lumbar spine were also more prevalent at 26.6% in those aged ≥ 65, compared to 18.9% of those younger than 65. 5.0% of all admissions were for a nonserious musculoskeletal condition beyond the lumbar spine.

## Discussion

### Statement of principal findings

Over half the admissions from ED with a provisional diagnosis of nonserious back pain had an equivalent discharge diagnosis and so are likely to be suitable for a virtual hospital model of care. However, a significant proportion of patients admitted with nonserious back pain were subsequently diagnosed with a specific pathology likely unsuitable for virtual care; 14.2% with a serious spinal pathology and 23.9% with a serious pathology beyond the lumbar spine. The most common serious spinal pathologies were fracture (8.7%) and infection (2.1%), and the most common serious pathologies beyond the spine were pathological fracture (7.3%) and infection (4.3%). These results suggest that patient selection would be key to the successful implementation of a virtual hospital model of care as an alternative to inpatient admission for LBP.

### Strengths and weaknesses of the study

This is the first study to investigate discharge diagnoses in patients who presented to ED with back pain and were admitted as inpatients with a provisional diagnosis of nonserious LBP. A strength of the study is that we sourced hospital admission data over a 4.75-year period, with a large sample size drawn from three university teaching hospitals in Sydney. We do acknowledge that rates of specific pathologies may be different in other health districts. Detailed consideration of ICD-10-AM codes by two experienced clinicians increased the precision of the diagnostic data we extracted. However, ICD-10-AM codes are applied by administrative staff to diagnostic information in the electronic medical record, therefore there is potential for error in the initial clinical coding. We are unable to report on rates of serious pathology ‘red flags’, as this data was not extracted from medical records.

This study did not consider other criteria to judge a patient’s suitability for virtual care. We understand that comorbid health conditions, patients’ existing social support and ability to navigate virtual care, as well as preferences, would all influence whether a patient would be suitable. We plan to evaluate these issues in a separate series of studies.

### Comparison to other studies

An Australian study of 712 admissions via ED to general medical or rheumatology wards over 36-months [12] found a similar patient profile for nonserious back pain admissions, with a median age of 67, 63% female patients, staying a median of 4[IQR 2–8] days. However, they found 81% of their cohort to have ‘nonserious back pain,’ in contrast to our rate of 57%. They also found a vertebral fracture prevalence of 14%, in contrast to 9% in our study. Rates of serious spinal and non-lumbar pathologies are understandably different between our studies as patients requiring surgical intervention for cord compression, cauda equina syndrome, or non-musculoskeletal back pain were excluded from the Kyi et al. study. Our study collected data from all hospital units, whereas Kyi et al. reported admissions to general medical and rheumatology units only. An important consideration is that comorbidities were present in 78% of their cohort, including 16% with osteoporosis and 10% with malignancies. These comorbidities may well impact suitability for virtual admission.

### Implications for clinicians and policymakers

Our results would suggest that up to 57% of ED LBP patients who are currently admitted as an inpatient may be suitable for virtual hospital care, which is ~ 250 patients per year. We have previously shown that each of these admissions typically costs ~ $A15,000 [13], meaning that virtualising admission for these patients could result in significant infrastructure and healthcare cost savings.

### Unanswered questions and future research

A key clinical challenge in implementing virtual hospital care is the differential diagnosis of nonserious, serious spinal, and non-lumbar pathologies in those presenting to ED with back pain. Protocols need to be developed to reduce the risk of patients being admitted to virtual hospitals with serious pathology as the cause of their back pain. Transfer to virtual care from ED short-stay units or the inpatient ward, once laboratory tests and imaging results are available to confirm nonserious back pain, maybe a suitable clinical pathway. We plan to evaluate the implementation of a virtual hospital model of back pain in the Sydney Local Health District in 2022.

## Supplementary Information

Below is the link to the electronic supplementary material.

Supplementary file1 (DOCX 32 kb)
