# Supplementary material for: Many people admitted to hospital with a provisional diagnosis of nonserious back pain are subsequently found to have serious pathology as the underlying cause
Source: Clin Rheumatol. 2022 Jan 11;41(6):1867–71. doi: 10.1007/s10067-022-06054-w (PMC9119888; doi:10.1007/s10067-022-06054-w)
Supplement: Supplementary file 1 — Supplementary file1 (DOCX 32 kb) [file 10067_2022_6054_MOESM1_ESM.docx]

**ICD-10-AM code groupings for discharge diagnosis**

| **Non-specific LBP** |
| --- |
| M41.96 [2] - Unspecified scoliosis, lumbar region |
| M43.04 [2] - Spondylolysis, thoracic region |
| M43.05 [2] - Spondylolysis, thoracolumbar region |
| M43.16 [2] - Spondylolisthesis, lumbar region |
| M43.17 [2] - Spondylolisthesis, lumbosacral region |
| M47.84 [2] - Other spondylosis, thoracic region |
| M47.85 [2] - Other spondylosis, thoracolumbar region |
| M47.86 [2] - Other spondylosis, lumbar region |
| M47.87 [2] - Other spondylosis, lumbosacral region |
| M47.96 [2] - Unspecified spondylosis, lumbar region |
| M47.97 [2] - Unspecified spondylosis, lumbosacral region |
| M54.5 [2] - Low back pain |
| M54.6 [2] - Pain in thoracic spine |
| M54.84 [2] - Other dorsalgia, thoracic region |
| M54.85 [2] - Other dorsalgia, thoracolumbar region |
| M54.86 [2] - Other dorsalgia, lumbar region |
| M54.87 [2] - Other dorsalgia, lumbosacral region |
| M54.88 [2] - Other dorsalgia, sacral and sacrococcygeal region |
| M54.89 [2] - Other dorsalgia, site unspecified |
| M54.90 [2] - Unspecified dorsalgia, multiple sites in spine |
| M54.94 [2] - Unspecified dorsalgia, thoracic region |
| M54.95 [2] - Unspecified dorsalgia, thoracolumbar region |
| M54.96 [2] - Unspecified dorsalgia, lumbar region |
| M54.99 [2] - Unspecified dorsalgia, site unspecified |
| M79.79 [2] - Fibromyalgia, site unspecified |
| S30.81 [2] - Other superficial injuries of abdomen, lower back and pelvis, abrasion |
| S30.98 [2] - Superficial injury of abdomen, lower back and pelvis, part unspecified, other |
| S33.51 [2] - Sprain and strain of lumbosacral [joint] [ligament] |
| S33.7 [2] - Sprain and strain of other and unspecified parts of lumbar spine and pelvis |
| S39.0 [2] - Injury of muscle and tendon of abdomen, lower back and pelvis |
| S39.8 [2] - Other specified injuries of abdomen, lower back and pelvis |
| S39.9 [2] - Unspecified injury of abdomen, lower back and pelvis |
| **Radicular back pain** |
| G14 [2] - Postpolio syndrome |
| G54.4 [2] - Lumbosacral root disorders, not elsewhere classified |
| G54.9 [2] - Nerve root and plexus disorder, unspecified |
| G55.1 [2] - Nerve root and plexus compressions in intervertebral disc disorders (M50-M51+) |
| G55.3 [2] - Nerve root and plexus compressions in other dorsopathies (M45-M46+, M48.-+, M53-M54+) |
| G57.1 [2] - Meralgia paraesthetica |
| M47.26 [2] - Other spondylosis with radiculopathy, lumbar region |
| M47.27 [2] - Other spondylosis with radiculopathy, lumbosacral region |
| M48.00 [2] - Spinal stenosis, multiple sites in spine |
| M48.05 [2] - Spinal stenosis, thoracolumbar region |
| M48.06 [2] - Spinal stenosis, lumbar region |
| M48.07 [2] - Spinal stenosis, lumbosacral region |
| M51.1 [2] - Lumbar and other intervertebral disc disorders with radiculopathy (G55.1*) |
| M51.2 [2] - Other specified intervertebral disc displacement |
| M51.3 [2] - Other specified intervertebral disc degeneration |
| M51.8 [2] - Other specified intervertebral disc disorders |
| M54.16 [2] - Radiculopathy, lumbar region |
| M54.17 [2] - Radiculopathy, lumbosacral region |
| M54.18 [2] - Radiculopathy, sacral and sacrococcygeal region |
| M54.3 [2] - Sciatica |
| M54.4 [2] - Lumbago with sciatica |
| S33.0 [2] - Traumatic rupture of lumbar intervertebral disc |
| S34.2 [2] - Injury of nerve root of lumbar and sacral spine |
| **Other musculoskeletal condition (non-lumbar)** |
| M10.96 [2] - Gout, unspecified, lower leg |
| M11.28 [2] - Other chondrocalcinosis, other site |
| M13.86 [2] - Other specified arthritis, lower leg |
| M13.98 [2] - Arthritis, unspecified, other site |
| M16.0 [2] - Primary coxarthrosis, bilateral |
| M16.1 [2] - Other primary coxarthrosis |
| M17.0 [2] - Primary gonarthrosis, bilateral |
| M17.1 [2] - Other primary gonarthrosis |
| M19.07 [2] - Primary arthrosis of other joints, ankle and foot |
| M19.08 [2] - Primary arthrosis of other joints, other site |
| M23.22 [2] - Derangement of posterior horn of medial meniscus due to old tear or injury |
| M25.51 [2] - Pain in a joint, shoulder region |
| M25.55 [2] - Pain in a joint, pelvic region and thigh |
| M25.85 [2] - Other specified joint disorders, pelvic region and thigh |
| M25.95 [2] - Unspecified joint disorder, pelvic region and thigh |
| M25.98 [2] - Unspecified joint disorder, other site |
| M35.3 [2] - Polymyalgia rheumatica |
| M46.92 [2] - Unspecified inflammatory spondylopathy, cervical region |
| M47.12 [2] - Other spondylosis with myelopathy, cervical region |
| M48.02 [2] - Spinal stenosis, cervical region |
| M50.0 [2] - Cervical disc disorder with myelopathy |
| M50.1 [2] - Cervical disc disorder with radiculopathy |
| M53.82 [2] - Other specified dorsopathies, cervical region |
| M54.12 [2] - Radiculopathy, cervical region |
| M54.2 [2] - Cervicalgia |
| M54.81 [2] - Other dorsalgia, occipito-atlanto-axial region |
| M62.65 [2] - Muscle strain, pelvic region and thigh |
| M65.93 [2] - Unspecified synovitis and tenosynovitis, forearm |
| M70.6 [2] - Trochanteric bursitis |
| M71.38 [2] - Other bursal cyst, other site |
| M71.95 [2] - Unspecified bursopathy, pelvic region and thigh |
| M75.1 [2] - Rotator cuff syndrome |
| M76.0 [2] - Gluteal tendinitis |
| M76.8 [2] - Other enthesopathies of lower limb, excluding foot |
| M79.16 [2] - Myalgia, lower leg |
| M79.18 [2] - Myalgia, other site |
| M79.65 [2] - Pain in limb, pelvic region and thigh |
| M79.66 [2] - Pain in limb, lower leg |
| M79.85 [2] - Other specified soft tissue disorders, pelvic region and thigh |
| M81.81 [2] - Other osteoporosis, shoulder region |
| M81.98 [2] - Unspecified osteoporosis, other site |
| M84.35 [2] - Stress fracture, not elsewhere classified, pelvic region and thigh |
| **Cauda equina, myelopathy or spinal cord compression/injury** |
| G83.4 [2] - Cauda equina syndrome |
| G95.2 [2] - Cord compression, unspecified |
| G95.8 [2] - Other specified diseases of spinal cord |
| M21.37 [2] - Wrist or foot drop (acquired), ankle and foot |
| M47.16 [2] - Other spondylosis with myelopathy, lumbar region |
| M47.17 [2] - Other spondylosis with myelopathy, lumbosacral region |
| M51.0 [2] - Lumbar and other intervertebral disc disorders with myelopathy |
| R20.8 [2] - Other and unspecified disturbances of skin sensation |
| S24.10 [2] - Injury of thoracic spinal cord unspecified |
| S24.12 [2] - Incomplete cord syndrome of thoracic spinal cord |
| **Dislocation of thoracolumbar vertebrae** |
| S33.13 [2] - Dislocation of L3/L4 lumbar vertebrae |
| S33.14 [2] - Dislocation of L4/L5 lumbar vertebrae |
| S23.13 [2] - Dislocation of T5/T6 and T6/T7 thoracic vertebrae |
| **Fracture (spinal)** |
| S22.06 [2] - Fracture of thoracic vertebra, T11 and T12 level |
| S32.03 [2] - Fracture of lumbar vertebra, L3 level |
| S32.1 [2] - Fracture of sacrum |
| S22.02 [2] - Fracture of thoracic vertebra, T3 and T4 level |
| S22.03 [2] - Fracture of thoracic vertebra, T5 and T6 level |
| S22.04 [2] - Fracture of thoracic vertebra, T7 and T8 level |
| S22.05 [2] - Fracture of thoracic vertebra, T9 and T10 level |
| S22.06 [2] - Fracture of thoracic vertebra, T11 and T12 level |
| S32.00 [2] - Fracture of lumbar vertebra, level unspecified |
| S32.01 [2] - Fracture of lumbar vertebra, L1 level |
| S32.02 [2] - Fracture of lumbar vertebra, L2 level |
| S32.03 [2] - Fracture of lumbar vertebra, L3 level |
| S32.04 [2] - Fracture of lumbar vertebra, L4 level |
| S32.05 [2] - Fracture of lumbar vertebra, L5 level |
| S32.1 [2] - Fracture of sacrum |
| S32.2 [2] - Fracture of coccyx |
| S32.82 [2] - Fracture of lumbosacral spine, part unspecified |
| S32.01 [2] - Fracture of lumbar vertebra, L1 level |
| M48.48 [2] - Fatigue fracture of vertebra, sacral and sacrococcygeal region |
| **Infection (spinal)** |
| G06.1 [2] - Intraspinal abscess and granuloma |
| M46.22 [2] - Osteomyelitis of vertebra, cervical region |
| M46.24 [2] - Osteomyelitis of vertebra, thoracic region |
| M46.25 [2] - Osteomyelitis of vertebra, thoracolumbar region |
| M46.26 [2] - Osteomyelitis of vertebra, lumbar region |
| M46.27 [2] - Osteomyelitis of vertebra, lumbosacral region |
| M46.34 [2] - Infection of intervertebral disc (pyogenic), thoracic region |
| M46.36 [2] - Infection of intervertebral disc (pyogenic), lumbar region |
| M46.44 [2] - Unspecified discitis, thoracic region |
| M46.45 [2] - Unspecified discitis, thoracolumbar region |
| M46.46 [2] - Unspecified discitis, lumbar region |
| M46.47 [2] - Unspecified discitis, lumbosacral region |
| M46.56 [2] - Other infective spondylopathies, lumbar region |
| **Neurological condition (spinal)** |
| G95.8 [2] - Other specified diseases of spinal cord |
| G37.3 [2] - Acute transverse myelitis in demyelinating disease of central nervous system |
| **Neoplasm (Spinal)** |
| D43.4 [2] - Neoplasm of uncertain or unknown behaviour of spinal cord |
| **Osteoporotic fracture (spinal)** |
| M48.54 [2] - Collapsed vertebra, not elsewhere classified, thoracic region |
| M48.55 [2] - Collapsed vertebra, not elsewhere classified, thoracolumbar region |
| M48.56 [2] - Collapsed vertebra, not elsewhere classified, lumbar region |
| **Post-surgical or procedural complication** |
| M96.6 [2] - Fracture of bone following insertion of orthopaedic implant, joint prosthesis, or bone plate |
| T80.2 [2] - Infections following infusion, transfusion and therapeutic injection |
| T81.4 [2] - Wound infection following a procedure, not elsewhere classified |
| T81.83 [2] - Pain following a procedure, not elsewhere classified |
| T81.89 [2] - Other complications following a procedure, not elsewhere classified |
| T84.2 [2] - Mechanical complication of internal fixation device of other bones |
| T84.83 [2] - Pain following insertion of internal orthopaedic prosthetic devices, implants and grafts |
| T85.72 [2] - Infection and inflammatory reaction due to nervous system prosthetic devices, implants and grafts |
| T88.59 [2] - Complications of anaesthesia, not elsewhere classified |
| **Inflammatory spondylopathy** |
| M45.04 [2] - Ankylosing spondylitis, thoracic region |
| M45.06 [2] - Ankylosing spondylitis, lumbar region |
| M45.07 [2] - Ankylosing spondylitis, lumbosacral region |
| M46.1 [2] - Sacroiliitis, not elsewhere classified |
| M46.84 [2] - Other specified inflammatory spondylopathies, thoracic region |
| M46.86 [2] - Other specified inflammatory spondylopathies, lumbar region |
| M46.90 [2] - Unspecified inflammatory spondylopathy, multiple sites in spine |
| M46.96 [2] - Unspecified inflammatory spondylopathy, lumbar region |
| M46.97 [2] - Unspecified inflammatory spondylopathy, lumbosacral region |
| M48.14 [2] - Ankylosing hyperostosis [Forestier], thoracic region |
| M48.86 [2] - Other specified spondylopathies, lumbar region |
| **Cardiovascular condition** |
| D68.3 [2] - Haemorrhagic disorder due to circulating anticoagulants |
| I21.4 [2] - Acute subendocardial myocardial infarction |
| I25.9 [2] - Chronic ischaemic heart disease, unspecified |
| I26.9 [2] - Pulmonary embolism without mention of acute cor pulmonale |
| I42.8 [2] - Other cardiomyopathies |
| I48.0 [2] - Paroxysmal atrial fibrillation |
| I48.9 [2] - Atrial fibrillation and atrial flutter, unspecified |
| I50.0 [2] - Congestive heart failure |
| I70.0 [2] - Atherosclerosis of aorta |
| I71.4 [2] - Abdominal aortic aneurysm, without mention of rupture |
| I74.8 [2] - Embolism and thrombosis of other arteries |
| I80.41 [2] - Phlebitis and thrombophlebitis of superficial vessels of upper extremities |
| I95.10 [2] - Orthostatic hypotension, unspecified |
| I95.9 [2] - Hypotension, unspecified |
| T82.89 [2] - Other specified complications of cardiac and vascular prosthetic devices, implants and grafts |
| **Trauma (contusion, wound, or fracture beyond lumbar spine)** |
| L89.99 [2] - Pressure injury, unspecified stage, other site of lower extremity (excluding heel and toe) |
| S00.05 [2] - Superficial injury of scalp, contusion |
| S01.41 [2] - Open wound of cheek |
| S09.9 [2] - Unspecified injury of head |
| S19.9 [2] - Unspecified injury of neck |
| S22.32 [2] - Fracture of one rib, other than first rib |
| S22.42 [2] - Multiple rib fractures, involving two ribs |
| S27.0 [2] - Traumatic pneumothorax |
| S29.9 [2] - Unspecified injury of thorax |
| S32.4 [2] - Fracture of acetabulum |
| S32.5 [2] - Fracture of pubis |
| S50.0 [2] - Contusion of elbow |
| S52.00 [2] - Fracture of upper end of ulna, part unspecified |
| S73.18 [2] - Sprain and strain of other specified sites of hip |
| S76.3 [2] - Injury of muscle and tendon of the posterior muscle group at thigh level |
| S79.8 [2] - Other specified injuries of hip and thigh |
| S79.9 [2] - Unspecified injury of hip and thigh |
| S80.81 [2] - Abrasion of lower leg |
| S80.9 [2] - Superficial injury of lower leg, unspecified |
| S82.6 [2] - Fracture of lateral malleolus |
| S83.2 [2] - Tear of meniscus, current |
| S92.0 [2] - Fracture of calcaneus |
| S92.3 [2] - Fracture of metatarsal bone |
| T79.6 [2] - Traumatic ischaemia of muscle |
| **Delirium or dementia** |
| F01.9 [2] - Vascular dementia, unspecified |
| F03 [2] - Unspecified dementia |
| F05.0 [2] - Delirium not superimposed on dementia, so described |
| F05.1 [2] - Delirium superimposed on dementia |
| F05.8 [2] - Other delirium |
| F05.9 [2] - Delirium, unspecified |
| **Gastroenterological condition** |
| K29.30 [2] - Chronic superficial gastritis, without mention of haemorrhage |
| K29.70 [2] - Gastritis, unspecified, without mention of haemorrhage |
| K35.8 [2] - Acute appendicitis, other and unspecified |
| K51.9 [2] - Ulcerative colitis, unspecified |
| K56.5 [2] - Intestinal adhesions [bands] with obstruction |
| K59.0 [2] - Constipation |
| K66.1 [2] - Haemoperitoneum |
| K70.3 [2] - Alcoholic cirrhosis of liver |
| K76.9 [2] - Liver disease, unspecified |
| K80.00 [2] - Calculus of gallbladder with acute cholecystitis, without mention of obstruction |
| K80.10 [2] - Calculus of gallbladder with other cholecystitis, without mention of obstruction |
| K80.30 [2] - Calculus of bile duct with cholangitis, without mention of obstruction |
| K83.0 [2] - Cholangitis |
| K85.9 [2] - Acute pancreatitis, unspecified |
| **Infection (beyond spine)** |
| A08.4 [2] - Viral intestinal infection, unspecified |
| A09.0 [2] - Other gastroenteritis and colitis of infectious origin |
| A09.9 [2] - Gastroenteritis and colitis of unspecified origin |
| A17.0 [2] - Tuberculous meningitis |
| A41.0 [2] - Sepsis due to Staphylococcus aureus |
| A41.1 [2] - Sepsis due to other specified staphylococcus |
| A41.51 [2] - Sepsis due to Escherichia coli [E. Coli] |
| A41.58 [2] - Sepsis due to other Gram-negative organisms |
| A41.9 [2] - Sepsis, unspecified |
| A49.01 [2] - Staphylococcus aureus infection, unspecified site |
| A49.1 [2] - Streptococcal and enterococcal infection, unspecified site |
| B01.9 [2] - Varicella without complication |
| B02.9 [2] - Zoster without complication |
| B23.0 [2] - Acute HIV infection syndrome |
| B30.8 [2] - Other viral conjunctivitis |
| B34.1 [2] - Enterovirus infection, unspecified site |
| B34.8 [2] - Other viral infections of unspecified site |
| B34.9 [2] - Viral infection, unspecified |
| G03.8 [2] - Meningitis due to other specified causes |
| G03.9 [2] - Meningitis, unspecified |
| I33.0 [2] - Acute and subacute infective endocarditis |
| J10.0 [2] - Influenza with pneumonia, other influenza virus identified |
| J10.1 [2] - Influenza with other respiratory manifestations, other influenza virus identified |
| J18.9 [2] - Pneumonia, unspecified |
| J22 [2] - Unspecified acute lower respiratory infection |
| J44.0 [2] - Chronic obstructive pulmonary disease with acute lower respiratory infection |
| J47 [2] - Bronchiectasis |
| L02.2 [2] - Cutaneous abscess, furuncle and carbuncle of trunk |
| L02.3 [2] - Cutaneous abscess, furuncle and carbuncle of buttock |
| L03.13 [2] - Cellulitis of lower limb |
| M00.05 [2] - Staphylococcal arthritis and polyarthritis, pelvic region and thigh |
| M00.08 [2] - Staphylococcal arthritis and polyarthritis, other site |
| M60.05 [2] - Infective myositis, pelvic region and thigh |
| M60.08 [2] - Infective myositis, other site |
| M80.98 [2] - Unspecified osteoporosis with pathological fracture, other site |
| M86.18 [2] - Other acute osteomyelitis, other site |
| M86.65 [2] - Other chronic osteomyelitis, pelvic region and thigh |
| M86.68 [2] - Other chronic osteomyelitis, other site |
| M86.97 [2] - Unspecified osteomyelitis, ankle and foot |
| M90.28 [2] - Osteopathy in other infectious diseases classified elsewhere, other site |
| N39.0 [2] - Urinary tract infection, site not specified |
| O23.5 [2] - Infections of the genital tract in pregnancy |
| O86.0 [2] - Infection of obstetric surgical wound |
| O86.2 [2] - Urinary tract infection following delivery |
| T83.5 [2] - Infection and inflammatory reaction due to prosthetic device, implant and graft in urinary system |
| **Inflammatory arthropathy (beyond spine)** |
| M00.98 [2] - Pyogenic arthritis, unspecified, other site |
| M02.88 [2] - Other reactive arthropathies, other site |
| M06.95 [2] - Rheumatoid arthritis, unspecified, pelvic region and thigh |
| M08.95 [2] - Juvenile arthritis, unspecified, pelvic region and thigh |
| M10.95 [2] - Gout, unspecified, pelvic region and thigh |
| **Metabolic disorder** |
| D50.9 [2] - Iron deficiency anaemia, unspecified |
| E11.40 [2] - Type 2 diabetes mellitus with unspecified neuropathy |
| E11.41 [2] - Type 2 diabetes mellitus with diabetic mononeuropathy |
| E11.42 [2] - Type 2 diabetes mellitus with diabetic polyneuropathy |
| E46 [2] - Unspecified protein-energy malnutrition |
| E83.5 [2] - Disorders of calcium metabolism |
| E87.5 [2] - Hyperkalaemia |
| E87.7 [2] - Fluid overload |
| M62.59 [2] - Muscle wasting and atrophy, not elsewhere classified, site unspecified |
| M89.88 [2] - Other specified disorders of bone, other site |
| **Neurological condition (beyond spine)** |
| F11.3 [2] - Mental and behavioural disorders due to use of opioids, withdrawal state |
| F20.9 [2] - Schizophrenia, unspecified |
| F44.6 [2] - Dissociative anaesthesia and sensory loss |
| G20 [2] - Parkinson's disease |
| G21.0 [2] - Malignant neuroleptic syndrome |
| G31.8 [2] - Other specified degenerative diseases of nervous system |
| G32.0 [2] - Subacute combined degeneration of spinal cord in diseases classified elsewhere |
| G58.19 [2] - Complex regional pain syndrome type I, other specified site |
| G61.0 [2] - Guillain-Barre syndrome |
| G61.8 [2] - Other inflammatory polyneuropathies |
| G62.9 [2] - Polyneuropathy, unspecified |
| G90.2 [2] - Horner's syndrome |
| G93.8 [2] - Other specified disorders of brain |
| H81.1 [2] - Benign paroxysmal vertigo |
| I62.1 [2] - Nontraumatic extradural haemorrhage |
| I63.9 [2] - Cerebral infarction, unspecified |
| S06.02 [2] - Loss of consciousness of brief duration [less than 30 minutes] |
| **Leukaemia** |
| C91.10 [2] - Chronic lymphocytic leukaemia of B-cell type, without mention of remission |
| C91.11 [2] - Chronic lymphocytic leukaemia of B-cell type, in remission |
| C92.00 [2] - Acute myeloblastic leukaemia [AML], without mention of remission |
| C92.80 [2] - Acute myeloid leukaemia with multilineage dysplasia, without mention of remission |
| **Lymphoma** |
| C81.9 [2] - Hodgkin lymphoma, unspecified |
| C83.3 [2] - Diffuse large B-cell lymphoma |
| C83.7 [2] - Burkitt lymphoma |
| C85.9 [2] - Non-Hodgkin lymphoma, unspecified |
| C88.00 [2] - Waldenstrom macroglobulinaemia, without mention of remission |
| **Myeloma** |
| C90.00 [2] - Multiple myeloma, without mention of remission |
| **Neoplasm (beyond spine)** |
| C16.9 [2] - Malignant neoplasm of stomach, unspecified |
| C18.0 [2] - Malignant neoplasm of caecum |
| C19 [2] - Malignant neoplasm of rectosigmoid junction |
| C34.0 [2] - Malignant neoplasm of main bronchus |
| C34.1 [2] - Malignant neoplasm of upper lobe, bronchus or lung |
| C34.2 [2] - Malignant neoplasm of middle lobe, bronchus or lung |
| C34.3 [2] - Malignant neoplasm of lower lobe, bronchus or lung |
| C34.9 [2] - Malignant neoplasm of bronchus or lung, unspecified |
| C50.4 [2] - Malignant neoplasm of upper-outer quadrant of breast |
| C50.9 [2] - Malignant neoplasm of breast, unspecified part |
| C61 [2] - Malignant neoplasm of prostate |
| C77.2 [2] - Secondary and unspecified malignant neoplasm of intra-abdominal lymph nodes |
| C78.2 [2] - Secondary malignant neoplasm of pleura |
| C78.7 [2] - Secondary malignant neoplasm of liver and intrahepatic bile duct |
| C79.1 [2] - Secondary malignant neoplasm of bladder and other and unspecified urinary organs |
| C79.3 [2] - Secondary malignant neoplasm of brain and cerebral meninges |
| C79.5 [2] - Secondary malignant neoplasm of bone and bone marrow |
| C79.82 [2] - Secondary malignant neoplasm of genital organs |
| C79.88 [2] - Secondary malignant neoplasm of other specified sites |
| D27 [2] - Benign neoplasm of ovary |
| C49.9 [2] - Malignant neoplasm of connective and soft tissue, unspecified |
| **Gynaecological condition** |
| N83.1 [2] - Corpus luteum cyst |
| O72.2 [2] - Delayed and secondary postpartum haemorrhage |
| O99.8 [2] - Other specified diseases and conditions in pregnancy, childbirth and the puerperium |
| **Pathological fracture (beyond spine)** |
| M80.45 [2] - Drug-induced osteoporosis with pathological fracture, pelvic region and thigh |
| M80.48 [2] - Drug-induced osteoporosis with pathological fracture, other site |
| M80.85 [2] - Other osteoporosis with pathological fracture, pelvic region and thigh |
| M80.88 [2] - Other osteoporosis with pathological fracture, other site |
| M80.91 [2] - Unspecified osteoporosis with pathological fracture, shoulder region |
| M80.95 [2] - Unspecified osteoporosis with pathological fracture, pelvic region and thigh |
| M80.98 [2] - Unspecified osteoporosis with pathological fracture, other site |
| M84.45 [2] - Pathological fracture, not elsewhere classified, pelvic region and thigh |
| M84.48 [2] - Pathological fracture, not elsewhere classified, other site |
| M90.75 [2] - Fracture of bone in neoplastic disease, pelvic region and thigh (C00-D48+) |
| M90.78 [2] - Fracture of bone in neoplastic disease, other site |
| M90.79 [2] - Fracture of bone in neoplastic disease, site unspecified (C00-D48+) |
| S72.11 [2] - Fracture of intertrochanteric section of femur |
| **Respiratory condition** |
| J96.00 [2] - Acute respiratory failure, type I |
| J96.01 [2] - Acute respiratory failure, type II |
| J98.4 [2] - Other disorders of lung |
| **Urological condition** |
| N05.9 [2] - Unspecified nephritic syndrome, unspecified |
| N10 [2] - Acute tubulo-interstitial nephritis |
| N12 [2] - Tubulo-interstitial nephritis, not specified as acute or chronic |
| N13.2 [2] - Hydronephrosis with renal and ureteral calculous obstruction |
| N13.3 [2] - Other and unspecified hydronephrosis |
| N14.1 [2] - Nephropathy induced by other drugs, medicaments and biological substances |
| N17.9 [2] - Acute kidney failure, unspecified |
| N20.0 [2] - Calculus of kidney |
| N30.9 [2] - Cystitis, unspecified |
| N32.8 [2] - Other specified disorders of bladder |
| **Other pathology (eMR consulted for categorisation of admission)** |
| R07.4 [2] - Chest pain, unspecified |
| R10.1 [2] - Pain localised to upper abdomen |
| R10.2 [2] - Pelvic and perineal pain |
| R10.3 [2] - Pain localised to other parts of lower abdomen |
| R10.4 [2] - Other and unspecified abdominal pain |
| R11 [2] - Nausea and vomiting |
| R20.2 [2] - Paraesthesia of skin |
| R25.2 [2] - Cramp and spasm |
| R26.8 [2] - Other and unspecified abnormalities of gait and mobility |
| R29.6 [2] - Tendency to fall, not elsewhere classified |
| R29.88 [2] - Other and unspecified symptoms and signs involving the nervous system |
| R41.8 [2] - Other and unspecified symptoms and signs involving cognitive functions and awareness |
| R42 [2] - Dizziness and giddiness |
| R50.9 [2] - Fever, unspecified |
| R51 [2] - Headache |
| R52.0 [2] - Acute pain, not elsewhere classified |
| R52.2 [2] - Chronic pain |
| R53 [2] - Malaise and fatigue |
| R55 [2] - Syncope and collapse |
| R56.8 [2] - Other and unspecified convulsions |
| R60.0 [2] - Localised oedema |
| R79.89 [2] - Other specified abnormal findings of blood chemistry |
| R94.5 [2] - Abnormal results of liver function studies |
| T39.1 [2] - 4-Aminophenol derivatives |
| T39.3 [2] - Other nonsteroidal anti-inflammatory drugs [NSAID] |
| Z04.3 [2] - Examination and observation following other accident |
| Z74.2 [2] - Need for assistance at home and no other household member able to render care |
| T81.83 [2] - Pain following a procedure, not elsewhere classified |
| Z49.1 [2] - Extracorporeal dialysis |
